# Adenoid cystic carcinoma with cystic change of the parotid gland posing a diagnostic challenge: A rare case report

**DOI:** 10.1016/j.ijscr.2025.111974

**Published:** 2025-09-22

**Authors:** Seblewengel Maru Wubalem, Tamiru Kawucha Gelebo, Birhanu Kassie Reta, Mihret Adane Woldemichael, Sara Alemnew Wedaj, Shemsu Abraham Hussien

**Affiliations:** a Department of Pathology, Wachemo University, Hossana, Ethiopia; b Department of Pathology, Aksum University, Aksum, Ethiopia; c Department of ENT Surgery, Addis Ababa University, Addis Ababa, Ethiopia; d Department of Gynecology and Obstetrics, Wachemo University, Hossana, Ethiopia; e Department of Maxillofacial Surgery, Wachemo University, Hossana, Ethiopia

**Keywords:** Salivary gland neoplasms, Cystic lesions, Adenoid cystic carcinoma, Fine needle aspiration cytology, Diagnostic challenges, Case report

## Abstract

**Introduction and importance:**

Salivary gland neoplasms, comprising 2-6.5% of head and neck tumors, primarily arise in the parotid and submandibular glands. Salivary gland cystic lesions can be either neoplastic or non-neoplastic.

**Presentation of case:**

This is a 50-year-old male with a cystic adenoid cystic carcinoma (ACC) in the parotid gland, presenting with a right infraauricular swelling that had progressively enlarged over three years. Fine needle aspiration cytology (FNAC) indicated a benign cystic neoplasm. Histological examination, however, confirmed ACC with cystic changes.

**Clinical discussion:**

A cyst in salivary gland neoplasms can result from degeneration, necrosis, or hemorrhage within the tumor, or when the tumor arises within a non-neoplastic cyst. Differentiating between benign and malignant cystic salivary gland lesions is challenging preoperatively. Accurate preoperative diagnosis is essential to prevent unnecessary surgeries, and techniques such as ultrasound and FNAC are critical in assessing these lesions.

**Conclusion:**

Cystic changes in malignant tumors, particularly ACC, are not frequently reported, underlining the need for increased awareness among clinicians and pathologists. This case highlights the importance of including ACC in the differential diagnosis of cystic salivary gland tumors.

## Highlights

• **Salivary gland neoplasms are rare**, representing 2–6.5 % of head and neck neoplasms.
• Cystic salivary gland neoplasms are uncommon, and cystic adenoid cystic carcinoma is exceptionally rare.
• Cystic **salivary gland** neoplasms are a diagnostic challenge for both clinicians and pathologists
• Accurate preoperative diagnosis is crucial to avoid unnecessary surgeries and complications.

## 1 Introduction and importance

Salivary gland neoplasms represent 2–6.5 % of head and neck neoplasms. The most common site of involvement is the parotid gland, followed by the submandibular gland [1,2]. Cystic lesions of the salivary gland can be seen in both neoplastic (either benign or malignant) and non-neoplastic conditions [1,3]. The non-neoplastic cystic lesions mainly include obstructive sialadenopathy, lymphoepithelial cysts, sclerosing polycystic adenosis, polycystic disease, and epidermoid cysts. The neoplastic cystic salivary gland tumors comprise Warthin tumor, sebaceous adenoma, sebaceous lymphadenoma, intraductal papilloma, pleomorphic adenoma, mucoepidermoid carcinoma, acinic cell carcinoma, cystadenoma, cystadenocarcinoma, and secretory carcinoma [4]. Fine needle aspiration cytology (FNAC) and imaging studies such as ultrasonography (US) and magnetic resonance imaging (MRI) and are important diagnostic tools for differentiating benign and malignant cystic salivary gland lesions. This case report will present a cystic adenoid cystic carcinoma of the parotid gland in a 50-year-old male patient. ‘This case report has been reported in line with the SCARE checklist [13].

## 2 Presentation of case

A fifty-year-old male patient presented with a complaint of an occasional mildly painful, gradually increasing right infra-auricular swelling for three years. There was no associated paresthesia or motor deficits. There was no previous history of head and neck cancer surgery. He denies any constitutional symptoms, including fever, chills, night sweats, appetite changes, and weight loss. He has never drunk alcohol and has never smoked.

**Fig. 1:**
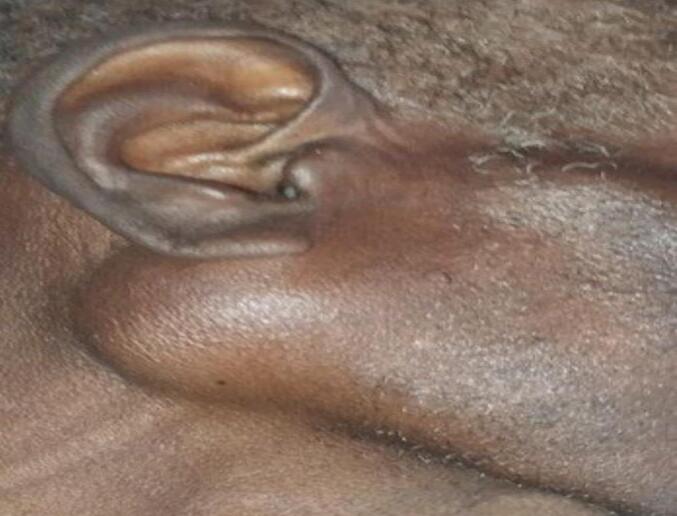
Physical examination: 3 × 4 cm right infraauricular swelling.

On maxillofacial examination, there is a swelling over the right infraauricular area that distorts his facial contour. Palpation reveals a well-circumscribed, freely movable, soft, 3 × 4 cm non-tender mass above the angle of the mandible (Fig. 1). There is no warmth, erythema, ulceration, or induration of the overlying soft tissue. Otherwise, the tympanic membranes are clear bilaterally. Cranial nerves II to XII are intact, with no weakness of the muscles of mastication. There is no sensory deficit in the V1-V3 distributions. The facial mimetic muscles are intact and symmetrical. The parotid papillae appear non-inflamed bilaterally, with clear saliva expressed from Stenson's duct. There are no mucosal ulcerations, intraoral extension of the mass or palpable submandibular or cervical lymphadenopathy.

**Fig. 2:**
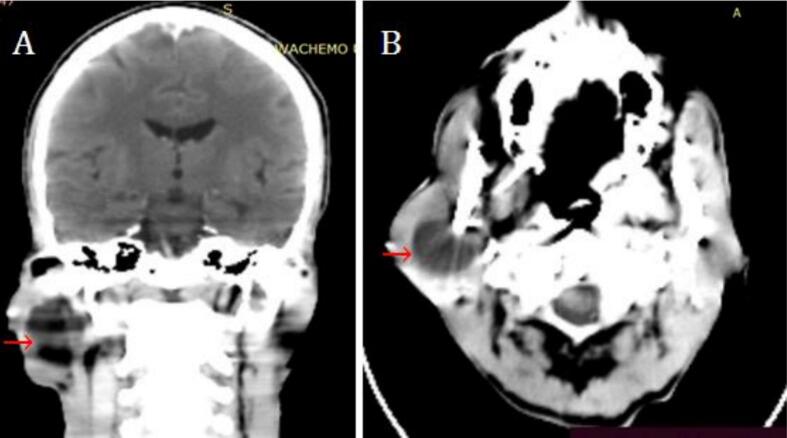
The CT scan shows cystic right parotid lesion (red arrows). (For interpretation of the references to colour in this figure legend, the reader is referred to the web version of this article.)

The patient was investigated with a head CT scan and fine needle aspiration cytology (FNAC). The CT scan showed a well-defined, fluid-attenuating right parotid gland lesion measuring 2.7 × 2.8 × 3.2 cm with smooth margins. No enhancing parts were seen (Fig. 2). On FNAC, 12 ml of straw-colored fluid was aspirated. The swelling collapsed after the aspiration and refilled immediately. Cytological examination revealed a few clusters of basaloid cells in a fluid background. Given these findings, it was diagnosed as a benign cystic neoplasm according to the Milan System for Reporting Salivary Gland Cytopathology (MSRSGC).

After written informed consent was obtained, the patient was transferred to the operating theatre, where a partial parotidectomy was performed through a modified Blair incision by a maxillofacial surgeon. Intraoperatively, a well-delineated 3 × 4 cm cystic mass was found on the tail of the parotid gland. Hemostasis was checked using the Valsalva maneuver, and a drainage tube was placed into the defect, away from the nerve. The drain was secured at the skin surface with a 2–0 silk suture, and the wound was closed in two layers and dressed. The patient was extubated safely and transferred to the post-anesthesia unit with stable vital signs. The specimen was sent for histopathological examination.

**Fig. 3:**
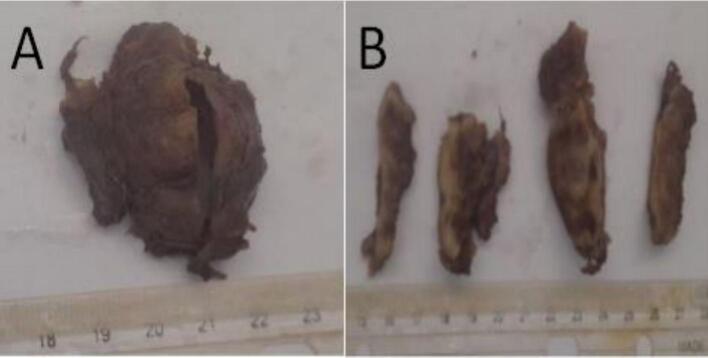
Gross picture: A well circumscribed cystic tissue (A) and a cut surface (B).

**Fig. 4:**
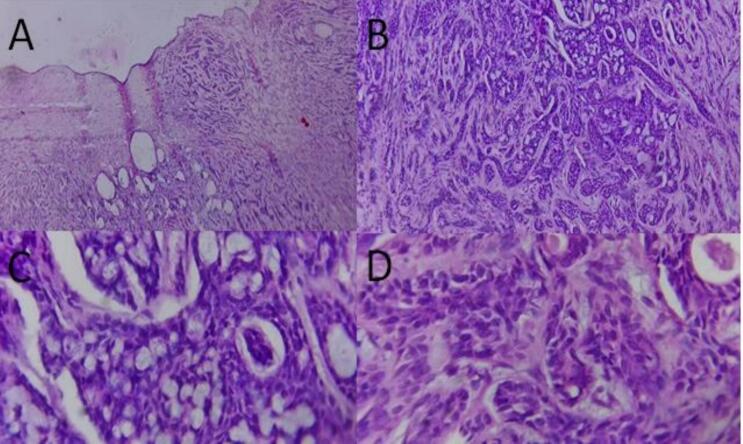
Histologic sections revealed a cyst wall lined with bilayered cuboidal cells (A, 10×). The wall is infiltrated with moderately pleomorphic, hyperchromatic, angulated basaloid cells arranged in solid nests (D, 40×) and a cribriform pattern (B, 20× and C, 40×)(hematoxylin and eosin).

On gross examination, a 3 × 3 cm partly opened, well-circumscribed cystic tissue was received. There was no any solid growth inside the cyst cavity (Fig. 3). On microscopic examination of hematoxylin and eosin-stained histologic sections revealed a cyst wall lined with bilayered cuboidal cells. The wall was infiltrated with moderately pleomorphic, hyperchromatic, angulated basaloid cells arranged in solid nests and a cribriform pattern. The small cystic spaces were filled with hyaine acellular materials. All resection margins were tumor-free, and there was no perineural invasion (Fig. 4). Based on these findings, a histological diagnosis of high grade adenoid cystic carcinoma was made.

Postoperatively, the patient was kept in the ward for close monitoring of the wound and for parenteral medications. The integrity of the facial nerve was evaluated in the immediate postoperative period. Antibiotic ointment was applied to the suture line during the initial postoperative care. The patient was discharged on the fifth postoperative day and showed no signs of metastasis or recurrence at the six-month follow-up.

## 3 Clinical discussion

Adenoid cystic carcinoma (ACC) is a rare malignancy arising from the secretory glands, most commonly involving the salivary glands, and it is most frequently observed in the 6th and 7th decades of life. It is the second most common malignant neoplasm of the major salivary glands and is also common in minor salivary gland [5]. Characteristically, it is an asymptomatic, slow-growing tumor; however, in late stages, pain and altered sensation may present due to perineural invasion. Perineural invasion is observed in around half of ACC cases [6].

A wide spectrum of neoplastic to non-neoplastic salivary gland pathologies may present as partially or completely cystic. Parotid gland cysts are rare, accounting for 2–5 % of parotid lesions [7]. A cyst in salivary gland neoplasms can result from degeneration, necrosis, or hemorrhage within the tumor [8], or when the tumor arises within a non-neoplastic cyst [9,10].

Accurate preoperative diagnosis is crucial to avoid unnecessary surgeries and their complications. US and FNAC are important diagnostic modalities for cystic salivary gland lesions [1,3,4,11]. The US features evaluated to reach a diagnosis include shape, margin, echogenicity, echotexture, posterior echo, and blood flow. However, the sensitivity is low, possibly because low-grade malignant tumors may exhibit US characteristics similar to those of benign tumors [1].

The sensitivity of FNAC in diagnosing cystic salivary gland lesions ranges from 41.6 % to 100 %. It is especially sensitive in diagnosing mucoepidermoid carcinoma in mucus-containing aspirates [4]. Most cystic salivary gland lesions yield paucicellular aspirates, which makes differentiation between benign and malignant tumors difficult. The diagnostic accuracy of the procedure can be enhanced by aspirating the residual solid focus after evacuating the cyst content, taking a detailed clinical history, and considering the broad differential diagnoses [8]. The cytologic findings of cystic salivary gland lesions are reported according to the MSRSGC. It has seven diagnostic categories, which include Non-diagnostic, Non-neoplastic, Atypia of Undetermined Significance, Benign Neoplasm, Salivary Gland Neoplasm of Uncertain Malignant Potential (SUMP), Suspicious for Malignancy, and Malignant [4,12]. The FNAC diagnosis in this case was a benign cystic neoplasm, based on the presence of a few clusters of basaloid cells in a fluid background. Reaching an accurate diagnosis by FNAC alone was difficult in our case since the aspirate consisted of hypo-cellular cystic fluid. However, upon examining the histological sections of the resected specimen, it was determined to be ACC with cystic changes. This highlights the difficulty of distinguishing between benign and malignant cystic tumors using cytology alone. In such scenarios, ultrasound-guided FNAC could be utilized to direct the aspiration from the solid focus and obtain adequate diagnostic material.

Although cystic changes are observed in a wide variety of malignant salivary gland tumors, cases with cystic changes in adenoid cystic carcinoma (ACC) have rarely been reported [1,3,4,7,11].

## 4 Conclusion

In conclusion, cystic salivary gland neoplasms are uncommon, and cystic ACC is exceptionally rare. These neoplasms pose a diagnostic challenge for both clinicians and pathologists, as there are a range of differential diagnoses and they yield paucicellular aspirates during FNAC. This case report alerts pathologists and clinicians to consider ACC in the differential diagnosis of cystic salivary gland tumors and include FNAC from the solid part of the cystic lesion in a diagnostic workup before any operative procedure.

## Abbreviations

ACC: Adenoid Cystic Carcinoma
FNAC: Fine Needle Aspiration Cytology
MRI: Magnetic Resonance Imaging
US: Ultrasonography
SUMP: Salivary Gland Neoplasm of Uncertain Malignant Potential
MSRSGC: Milan System for Reporting Salivary Gland Cytopathology

## Ethics approval

The study was notified to the university ethics committee; but this is case report and it does not need a specific ethical approval.

## Funding

This work did not receive any specific grant from funding agencies in the public, commercial, or not-for-profit sectors*.*

## Author contribution

**Seblewengel Maru Wubalem**: Conceptualization; Data curation; Resources; Visualization; writing – original draft; Writing – review and editing.

**Tamiru Kawucha Gelebo**: Resources; writing – original draft; Writing – review and editing.

**Birhanu Kassie Reta**: Data curation; Visualization; Writing – review and editing.

**Mihiret Adane Woldemichael**: Data curation; Visualization; Writing – review and editing.

**Sara Alemnew Wedaj**: Data curation; Visualization; Writing – review and editing.

**Shemsu Abraham Hussien**: Data curation; Supervision; Visualization; Writing – original draft; Writing – review and editing.

## Guarantor

Seblewengel Maru Wubalem

## Research registration number

N/A

## Consent for publication

Written informed consent was obtained from the patient for publication of this case report and accompanying images.

## Declaration of competing interest

The authors declare that they have no known competing financial interests or personal relationships that could have appeared to influence the work reported in this paper.
